# Contrast-enhanced ultrasound and shear wave elastography in the diagnosis of ACR TI-RADS 4 and 5 category thyroid nodules coexisting with Hashimoto’s thyroiditis

**DOI:** 10.3389/fonc.2022.1022305

**Published:** 2023-01-11

**Authors:** Bin Wang, Xiaoyan Ou, Juan Yang, Haibo Zhang, Xin-Wu Cui, Christoph F. Dietrich, Ai-Jiao Yi

**Affiliations:** ^1^ Department of Medical Ultrasound, Yueyang Central Hospital, Yueyang, China; ^2^ Department of Medical Ultrasound, Tongji Hospital, Tongji Medical College, Huazhong University of Science and Technology, Wuhan, China; ^3^ Department Allgemeine Innere Medizin, Kliniken Hirslanden Beau Site, Salem und Permanence, Bern, Switzerland

**Keywords:** shear wave elastography, Hashimoto’s thyroiditis, thyroid nodule, American College of Radiology, thyroid imaging reporting and data system, contrast-enhanced ultrasound

## Abstract

**Objective:**

This study aims to evaluate the value of contrast-enhanced ultrasound (CEUS), shear wave elastography (SWE), and their combined use in the differentiation of American College of Radiology (ACR) thyroid imaging reporting and data system (TI-RADS) 4 and 5 category thyroid nodules coexisting with Hashimoto’s thyroiditis (HT).

**Materials and methods:**

A total of 133 pathologically confirmed ACR TI-RADS 4 and 5 category nodules coexisting with HT in 113 patients were included; CEUS and SWE were performed for all nodules. The sensitivity, specificity, negative predictive value (NPV), positive predictive value (PPV), accuracy, and the area under the receiver operating characteristic curve (AUC) of the SWE, CEUS, and the combined use of both for the differentiation of benign and malignant nodules were compared, respectively.

**Results:**

Using CEUS alone, the sensitivity, specificity, PPV, NPV, and accuracy were 89.2%, 66.0%, 81.3%, 78.6%, and 80.5%, respectively. Using SWE alone, Emax was superior to Emin, Emean, and Eratio for the differentiation of benign and malignant nodules with the best cutoff Emax >46.8 kPa, which had sensitivity of 65.1%, specificity of 90.0%, PPV of 91.5%, NPV of 60.8%, and accuracy of 74.4%, respectively. Compared with the diagnostic performance of qualitative CEUS or/and quantitative SWE, the combination of CEUS and SWE had the best sensitivity, accuracy, and AUC; the sensitivity, specificity, PPV, NPV, accuracy, and AUC were 94.0%, 66.0%, 82.1%, 86.8%, 83.5%, and 0.80 (95% confidence interval: 0.713, 0.886), respectively.

**Conclusion:**

In conclusion, CEUS and SWE were useful for the differentiation of benign and malignant ACR TI-RADS 4 and 5 category thyroid nodules coexisting with HT. The combination of CEUS and SWE could improve the sensitivity and accuracy compared with using CEUS or SWE alone. It could be a non-invasive, reliable, and useful method to differentiate benign from malignant ACR TI-RADS 4 and 5 category thyroid nodules coexisting with HT.

## 1 Introduction

Thyroid nodules are a common disease and have shown a prevalence of 5% to 7% in the adult population with physical examination alone, while ultrasound examination shows a prevalence of 20% to 76% in this same population (1–3). Thyroid nodules are the most commonly found tumors in the cervical region, with nearly 10% being malignant nodules (4). It has been reported that one-third of thyroid cancer cases are prone to lymph node metastasis; thus, precise diagnosis and early treatment are significantly important for the recovery and better outcome of patients (5).

Hashimoto’s thyroiditis (HT) is the most common autoimmune thyroid disease, and the link between HT and thyroid cancer has been debated and remains controversial (6). The diagnosis of thyroid nodules with HT may be confusing in clinical work. Conventional ultrasound can differentiate benign from malignant nodules based on some characteristics, including marked hypo-echogenicity, a shape which is taller than wide, irregular margin, and micro-calcification (7). However, these characteristics have overlaps between benign and malignant nodules, especially those coexisting with HT. Some studies found that the irregular or micro-lobulated margins of benign nodules were more frequent in HT patients with a heterogeneous echogenicity background, and some conventional ultrasound characteristics were difficult to identify in the heterogeneous thyroid gland coexisting with HT, such as margin and calcification (8, 9). Thus, it is important to find a method to differentiate benign from malignant nodules coexisting with HT.

Currently, fine-needle aspiration (FNA) is the most effective and practical method used to reach a definitive diagnosis (10). However, most nodules are benign, and some malignant nodules frequently present an indolent behavior; therefore, not all thyroid nodules need FNA. To identify the most clinically significant malignant nodules and reduce the number of biopsies, the American College of Radiology (ACR) presents the thyroid imaging reporting and data system (TI-RADS) (7). However, the cancer risk levels were 5%–20% for ACR TI-RADS 4 category and at least 20% for ACR TI-RADS 5 category (11). There was a great overlap between benign and malignant nodules in ACR TI-RADS 4 and 5 categories, especially for nodules coexisting with TH. Thus, it is important to find a reliable and noninvasive method to differentiate benign from malignant ACR TI-RADS 4 and 5 category nodules, especially for nodules without recommendation of FNA.

In recent years, shear wave elastography (SWE) and contrast-enhanced ultrasound (CEUS) have been widely used in the diagnosis of thyroid nodules. SWE can quantitatively measure real-time tissue elasticity, along with a color-coded elasticity map (12). Many studies have found that the elasticity index of thyroid cancer is higher than that of benign thyroid nodules (13–17). CEUS can qualitatively or quantitatively evaluate the macro- and micro-vascularization patterns of thyroid nodules compared with the surrounding tissue, which is a promising noninvasive method for differentiating benign from malignant nodules. However, there were overlapping parameters and patterns with CEUS qualitative and quantitative evaluation in the differentiation of benign and malignant nodules, which indicate a limitation in the interpretation of tumor vascularization (18).

ACR TI-RADS 4 and 5 category thyroid nodules coexisting with HT were difficult to differentiate, and using CEUS or SWE alone had its limitation. The study aimed to explore the diagnostic performance of SWE, CEUS, and the combination of CEUS and SWE in ACR TI-RADS 4 and 5 category thyroid nodules coexisting with HT and find the reliable and noninvasive method to differentiate benign from malignant nodules, which would be beneficial to manage patients and improve their prognosis.

## 2 Materials and methods

This prospective study was approved by the ethics committee of Yueyang Central Hospital.

### 2.1 Patients

From April 2020 to July 2021, a total of 113 patients with 133 ACR TI-RADS 4 and 5 category thyroid nodules were recruited. The inclusion criteria were listed as follows: (a) patients aged 18 years or older with at least a thyroid nodule detected on the conventional ultrasound, (b) all patients gave signed informed consent before the SWE and CEUS examinations, (c) the pathology of all the thyroid nodules was confirmed *via* surgery according to standard clinical protocols, and (d) all patients were pathologically diagnosed as HT. Patients were excluded if they previously had a FNA, previously had radiofrequency ablation, had a contraindication of CEUS, or had unsatisfactory images.

### 2.2 Ultrasound examination

All ultrasound examinations including conventional ultrasound, SWE, and CEUS were performed with a high frequency transducer (L15-4 or L10-5 Aixplorer, Supersonic Imaging, France).

Conventional ultrasound was performed. When a target thyroid nodule was detected, the general characteristics were observed, including composition, echogenicity, shape, margin, and echogenic foci (7). Each lesion was classified into ACR TI-RADS 4 category (moderately suspicious) or ACR TI-RADS 5 category (highly suspicious).

The SWE imaging examinations were induced by the L10-5 transducer. In order to overcome the effect of artery pulsation on SWE measurement, a longitudinal section is often selected to conduct the SWE imaging. The probe was lightly applied while the patients were asked to hold their breath. The region of interest included the whole thyroid nodules, and in order to obtain satisfactory SWE imaging, several tips were suggested: (1) the upper edge of the region of interest is more than 1 cm away from the skin, (2) the depth of the lower edge of the region of interest should not exceed 4 cm, and (3) the length of the region of interest is two to three times larger than the nodules. The stiffness range of color map was from blue to red (0–180 kPa); the standard SWE imaging was obtained with several seconds of immobilization. The SWE measurement used quantification box (Q-box), and the Q-box should contain the nodules, excluding the surrounding organizations, which can automatically obtain Young’s modulus of Emin, Emean, and Emax. Eratio was defined as the ratio of Young’s modulus, which was obtained from the ratio of two Q-box in the same depth. The first Q-box was placed in the hardest region of the nodules, while the second Q-box was placed in the surrounding normal thyroid tissues. The diameter of the Q-box was 2 mm. The median was taken for five measurements to obtain more accurate results.

After the SWE imaging examination, CEUS was performed by using the L10-5 transducer. The patients were asked to lie down in the supine position, and the double-contrast mode was used to display nodules clearly. To avoid micro-bubble disruption, the focus was placed slightly deeper than the nodules with a low mechanical index that ranged from 0.06 to 0.08. A total of 25 mg sulfur hexafluoride (SonoVue^®^, Bracco International, Milan, Italy) was dissolved in 5 ml 0.9% sodium chloride and was injected with an intravenous bolus of 2.4 ml per patient, followed by a 5-ml saline flush. Each contrast imaging acquisition that lasted at least 120 s was stored in the machine hard disk.

All sonographic examinations were performed by the same investigator who had more than 10 years of experience in thyroid ultrasound and 5 years of experience in SWE and CEUS.

### 2.3 Statistical analysis

SPSS 23.0 and MedCalc 19.0 were used for all statistical analyses. The parameters of SWE and the enhancement patterns of CEUS were compared with the *t*-test, Kappa analysis, or Fisher’s exact test. A receiver operating characteristic (ROC) curve differentiating benign from malignant thyroid nodules was drawn according to Young’s modulus for each nodule. The optimal cutoff value and area under the curve (AUC) were calculated. The sensitivity, specificity, positive predictive value (PPV), negative predictive value (NPV), accuracy, and AUC of SWE, CEUS, or the combination of SWE and CEUS were calculated and compared, respectively. *P <*0.05 was considered to indicate statistical significance.

## 3 Results

### 3.1 General information between the benign and malignant nodules

**Figure 1:**
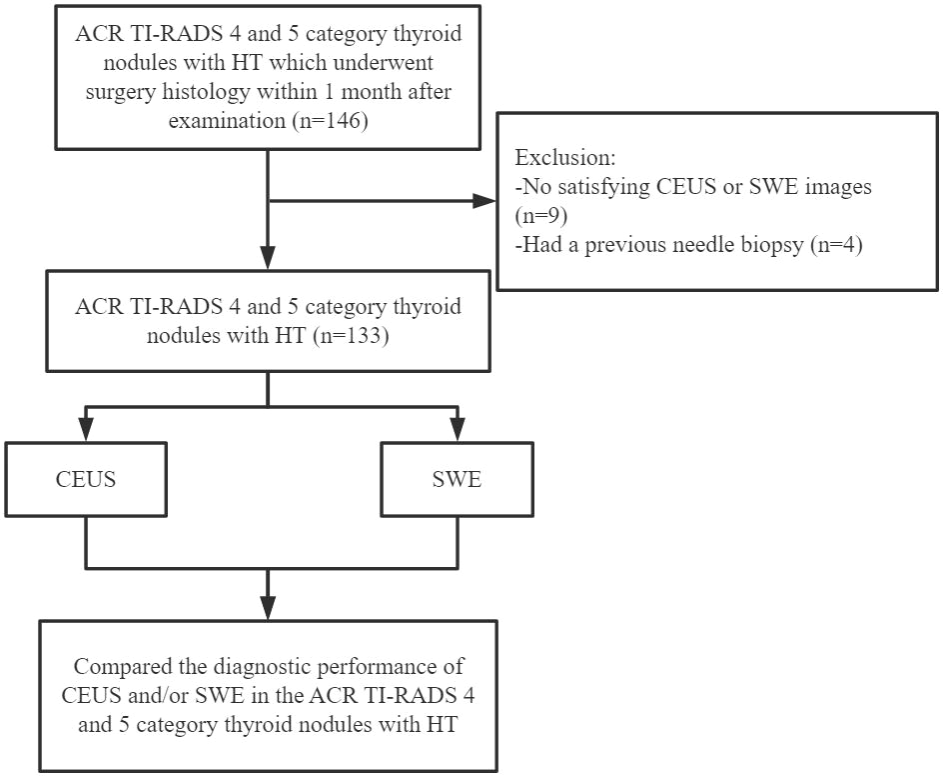
Flow chart for the selection of thyroid nodules.

Of the 133 nodules, the surgical pathological results showed that 50 nodules were benign and 83 nodules were malignant; all nodules were coexisting with HT (**Figure 1**). There was no significant difference in sex, age, the maximum diameter of nodules, and the number of nodules between benign and malignant nodules (*P* >0.05) (**Table 1**).

**Table 1:** General information between benign and malignant nodules.

Group	Case	Sex		Age	Number of nodules		Size
		Male	Female		Solitary nodule	Multiple nodules	
Benign group	50	6	44	46.16 ± 11.71	19	31	9.00 ± 5.56
Malignant group	83	13	70	46.21 ± 10.57	34	49	7.82 ± 3.27
*χ* ^2^/Fisher/*t*		0.342		0.485	0.114		1.540
*P*-value		0.559		0.629	0.735		0.125

### 3.2 CEUS in the differentiation of the benign and malignant nodules

The enhancement patterns of all thyroid nodules are summarized in **Table 2**. There were significant differences in peak enhancement, enhancement evenness, and ring enhancement with qualitative CEUS between benign and malignant thyroid nodules (*P* < 0.05). The benign thyroid nodules mostly manifested no enhancement, hyper-enhancement, iso-enhancement, homogeneous enhancement, or ring enhancement, while the malignant thyroid nodules mostly manifested hypo-enhancement, heterogeneous enhancement, or without ring enhancement. Using CEUS alone, the sensitivity, specificity, PPV, NPV, and accuracy were 89.2%, 66.0%, 81.3%, 78.6%, and 80.5%, respectively.

**Table 2:** Enhancement patterns of all thyroid nodules.

Group	Case	Peak intensity			Enhancement evenness		Ring enhancement	
		No enhancement or hyper-enhancement	Iso-enhancement	Hypo-enhancement	Homogeneous	Heterogeneous	With	Without
Benign	50	17	16	17	32	18	10	40
Malignant	83	1	10	72	7	76	1	82
*χ* ^2^/Fisher		44.124			46.487		–	
*P*-value		0.000			0.000		0.000	

### 3.3 SWE in the differentiation of the benign and malignant nodules

A ROC curve was drawn based on Emin, Emean, Emax, and Eratio to determine the optimal cutoff point for discriminating benign from malignant nodules. The optimal cutoff point was 27.8 kPa for Emin, 34.1 kPa for Emean, 46.8 kPa for Emax, and 1.25 for Eratio, respectively. Compared with the diagnostic performance of Emin, Emean, Emax, and Eratio (**Table 3**), Emax was superior to Emin, Emean, and Eratio, which had sensitivity of 65.1%, specificity of 90.0%, PPV of 91.5%, NPV of 60.8%, and accuracy of 74.4%.

**Table 3:** Diagnostic performance of Emin, Emean, Emax, and Eratio in differentiating benign and malignant nodules.

Elastography	Pathology	Benign	Malignant	Sensitivity	Specificity	Positive predictive value	Negative predictive value	Accuracy
Emin	Benign	47	3	45.80%	94.0%	92.7%	51.1%	63.9%
	Malignant	45	38					
Emean	Benign	41	9	62.7%	82.0%	85.2%	56.9%	69.6%
	Malignant	31	52					
Emax	Benign	45	5	65.1%	90.0%	91.5%	60.8%	74.4%
	Malignant	29	54					
Eratio	Benign	30	20	79.5%	60.0%	76.7%	63.8%	72.2%
	Malignant	17	66					

### 3.4 Comparing the diagnostic performance of CEUS, SWE, and the combination of CEUS and SWE in differentiating benign and malignant nodules

**Figure 2:**
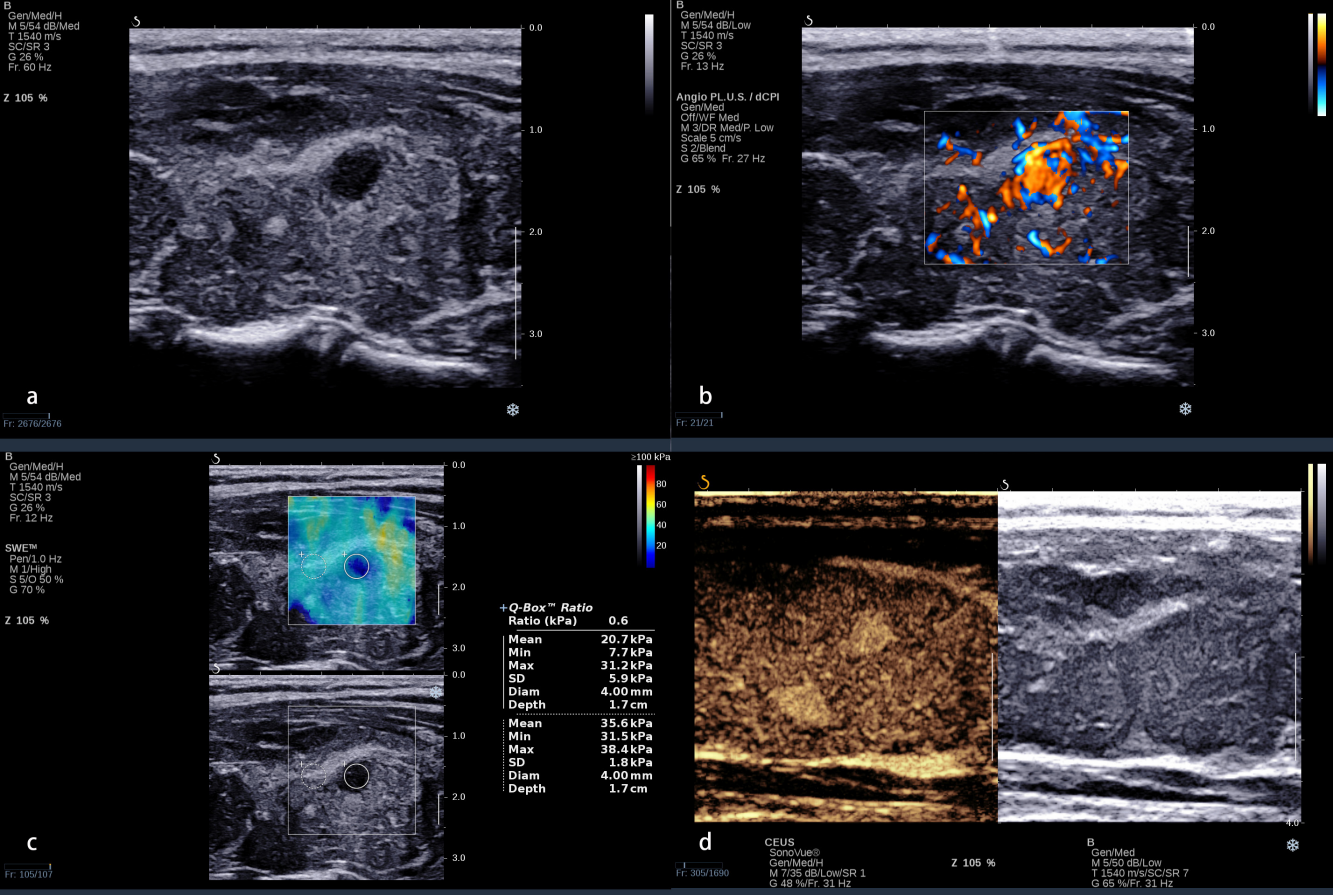
A 32-year-old woman with a nodule in the left lobe of the thyroid. **(A)** Conventional ultrasound revealed a 5 × 6-mm solid hypo-echoic nodule with a smooth margin, shape that was taller-than-wide, and without obvious calcification; this was categorized ACR TI-RADS 5. **(B)** The nodule presented a rich blood flow signal. **(C)** Quantitative shear wave elastography (SWE) revealed 7.7 kPa for Emin, 20.7 KPa for Emean, 31.2 kPa for Emax, and 0.6 for Eratio. **(D)** Qualitative contrast-enhanced ultrasound (CEUS) of the nodule revealed homogenous, hyper-enhancement. The combination of CEUS and SWE revealed that this nodule may be benign. The surgical pathology was focal Hashimoto’s thyroiditis.

**Figure 3:**
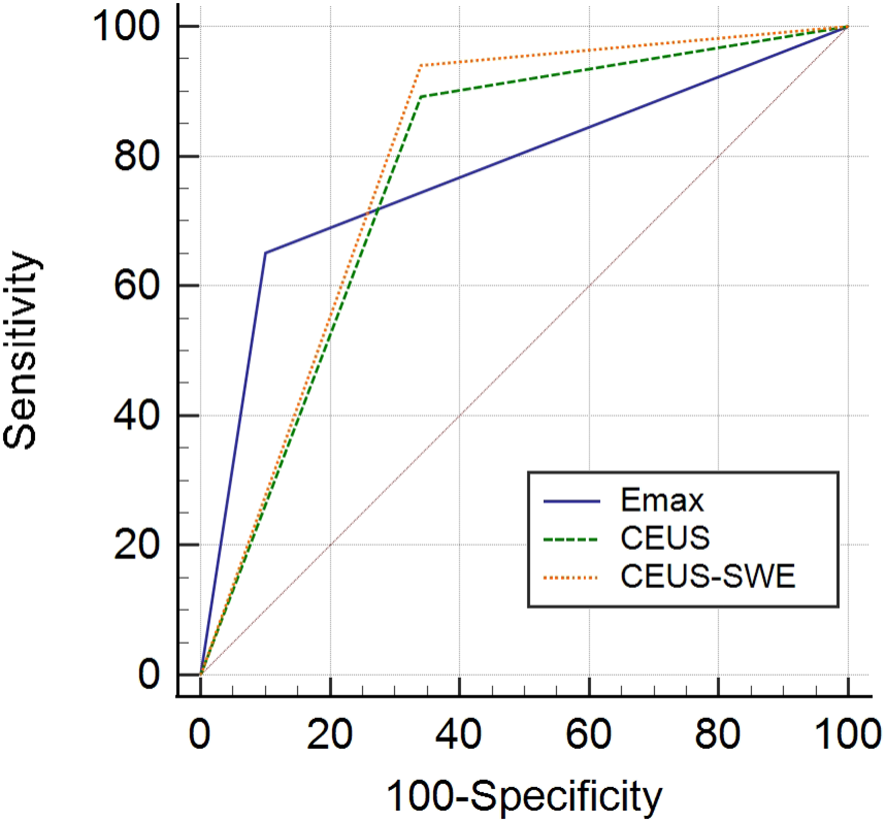
ROC of contrast-enhanced ultrasound (CEUS), shear wave elastography (SWE), and the combination of CEUS and SWE in differentiating benign and malignant nodules. ROC, receiver operating characteristic; AUC, area under the ROC curve.

The standard of the combination of qualitative CEUS and quantitative SWE was that the nodules were recognized as benign when qualitative CEUS manifested benign enhancement patterns and Emax <46.8 kPa simultaneously (**Figure 2**). Compared with using CEUS or SWE alone, combination of CEUS and SWE had the best sensitivity, accuracy, and AUC (**Figure 3**). The sensitivity, specificity, PPV, NPV, accuracy, and AUC were 94.0%, 66.0%, 82.1%, 86.8%, 83.5%, and 0.80 (95% confidence interval: 0.713, 0.886), respectively (**Table 4**).

**Table 4:** Diagnostic performance of contrast-enhanced ultrasound (CEUS), shear wave elastography (SWE), and the combination of CEUS and SWE in differentiating benign and malignant nodules.

	Sensitivity (%)	Specificity (%)	Positive predictive value (%)	Negative predictive value (%)	Accuracy (%)	Area under the curve	P*
CEUS	89.2	66.0	81.3	78.6	80.5	0.776 (0.687–0.864)	–
Emax	65.1	90.0	91.5	60.8	74.4	0.775 (0.695–0.856)	0.99
CEUS + Emax	94	66.0	82.1	86.8	83.5	0.800 (0.713–0.886)	0.04

Data are expressed as percentage (numbers). *Comparison of diagnostic performance of using CEUS alone with using SWE alone and the combination of CEUS and SWE.

### 3.5 Management and recommendation of ACR TI-RADS 4 and 5 category thyroid nodules coexisting with HT

According to the ACR TI-RADS, TI-RADS 4 category nodules with the maximal diameter ≧1.5 cm or TI-RADS 5 category nodules with the maximal diameter ≧1 cm were recommended for FNA. In this study, the malignant rate of TI-RADS 4 and 5 category nodules coexisting with HT and recommended for FNA was 50% (13/26), while the malignant rate of TI-RADS 4 and 5 category nodules coexisting with HT recommended for follow-up was 64.5% (70/107).

## 4 Discussion

Our study found that CEUS could evaluate tumor vascularization with enhancement patterns, and SWE could provide additional stiffness information, which was useful for the differentiation of ACR TI-RADS 4 and 5 category thyroid nodules coexisting with HT. The combination of CEUS and SWE could improve the sensitivity and accuracy compared with using CEUS or SWE alone.

ACR TI-RADS (7) presents a system for risk stratification of thyroid nodules, which was widely used to identify the most clinically significant malignant nodules and recommend them for FNA. According to the ACR TI-RADS, TI-RADS 4 category nodules with maximal diameter ≧1.5 cm or TI-RADS 5 category nodules with maximal diameter ≧1 cm were recommended for FNA; however, small thyroid cancers with maximal diameter ≤10 mm might mainly cause the “epidemic” of thyroid carcinoma (19). In this study, many TI-RADS 4 or 5 category nodules coexisting with HT and recommended for follow-up might be malignant. The malignant rate accounted for 65.4%. These small thyroid carcinomas made the patients endure a great psychological burden. Moreover, the ACR TI-RADS 4 and 5 category nodules coexisting with HT in patients were difficult to differentiate because of the heterogeneous and coarse thyroid parenchyma caused by the repetitive damage of chronic inflammation (20). Thus, it is a major challenge in the management of thyroid nodules with indeterminate cytology or suspicious conventional ultrasound features.

There were many studies on the qualitative CEUS features in differentiating benign thyroid nodules from malignant ones. According to the EFSUMB guidelines and recommendations for the clinical practice of contrast-enhanced ultrasound in non-hepatic applications (18), hypo-enhancement and heterogeneous enhancement are the predictors of malignancy on CEUS (21–26). A hypo-enhancement pattern was the most precise predictor of malignancy, which had sensitivity, specificity, and accuracy of 82%, 85%, and 84%, respectively (18, 23), while a heterogeneous enhancement pattern had sensitivity, specificity, and accuracy of 88.2%, 92.5%, and 90.4%, respectively (24–26). Some studies (27) found that a ring enhancement pattern was a very strong indicator of benign nodules, which had sensitivity, specificity, and accuracy of 83.0%, 94.1%, and 88.5%, respectively (24, 25). In this study, we observed CEUS enhancement patterns in ACR TI-RADS 4 and 5 category thyroid nodules coexisting with HT; it was consistent with a previous study. HT did not seem to affect the CEUS enhancement patterns in thyroid nodules.

When CEUS was used alone, 80.5% ACR TI-RADS 4 and 5 category nodules coexisting with HT could be accurately diagnosed. However, there were 34.0% (17/50) benign nodules that presented heterogeneous and hypo-enhancement, including 11 nodules that were nodular goiter with fibrosis or calcification, five nodules that were focal HT, and one nodule that was subacute thyroiditis. The malignant CEUS enhancement patterns of focal HT may be related to focal hypothyroidism with severe follicular degeneration (28), while that of subacute thyroiditis may be caused by the heterogeneous distribution of inflammatory cells with focal fiber hyperplasia. There were 10.8% (9/83) malignant nodules that present hyper-enhancement, iso-enhancement, or homogeneous enhancement, while the maximal diameter of 88.9% (eight out of nine) of these nodules was <10 mm, which may be because it was difficult to observe the enhancement patterns in small nodules or the neo-vascularization of some small thyroid nodules was not obvious. Therefore, using CEUS alone was insufficient to differentiate benign from malignant ACR TI-RADS 4 and 5 category nodules coexisting with HT.

In recent years, elastography has become available for thyroid nodule evaluation as reported in many studies, which is emerging as a potential method for the differentiation of benign and malignant thyroid nodules and may provide additional information to support clinical decision-making (29). Some studies (30–33) reported that strain elastography (SE) was useful for the prediction of malignancy and differentiation of thyroid nodules with indeterminate FNA cytology. Sengul et al. (31) found that SE score could affect the clinical decision-making for patients with indeterminate FNA cytology. Zhu et al. (32) reported that a high SE score was a significant predictor for malignancy. However, SE was challenged and criticized due to its operator dependency (34).

Compared with SE, SWE is less influenced by the experience and operation of the operator. Zhang et al. (35) found that SWE had high diagnostic efficiency for ACR TI-RADS 4 and 5 category thyroid nodules; the accuracy was 76.1%, with the best cutoff of Emax being 40.9 kPa. Chen et al. (36) conducted a meta-analysis with 4,296 thyroid nodules and found that Supersonic shear imaging showed high accuracy in the differentiation between benign and malignant thyroid nodules, which could serve as a noninvasive and important tool for thyroid nodule evaluation. Liao et al. (37) found that SWE could be an independent predictor for malignant thyroid nodules; the sensitivity and specificity were 81% and 65%, respectively, with the best cutoff of Emean being 32 kPa. In this study, we found that SWE was useful in the differentiation of benign and malignant ACR TI-RADS 4 and 5 category thyroid nodules, which was consistent with a previous study (35–37), and HT did not seem to affect the stiffness of thyroid nodules. Compared with Emin, Emean, and Eratio, Emax >46.8 kPa had the best diagnostic performance for ACR TI-RADS 4 and 5 category thyroid nodules coexisting with HT in this study.

**Figure 4:**
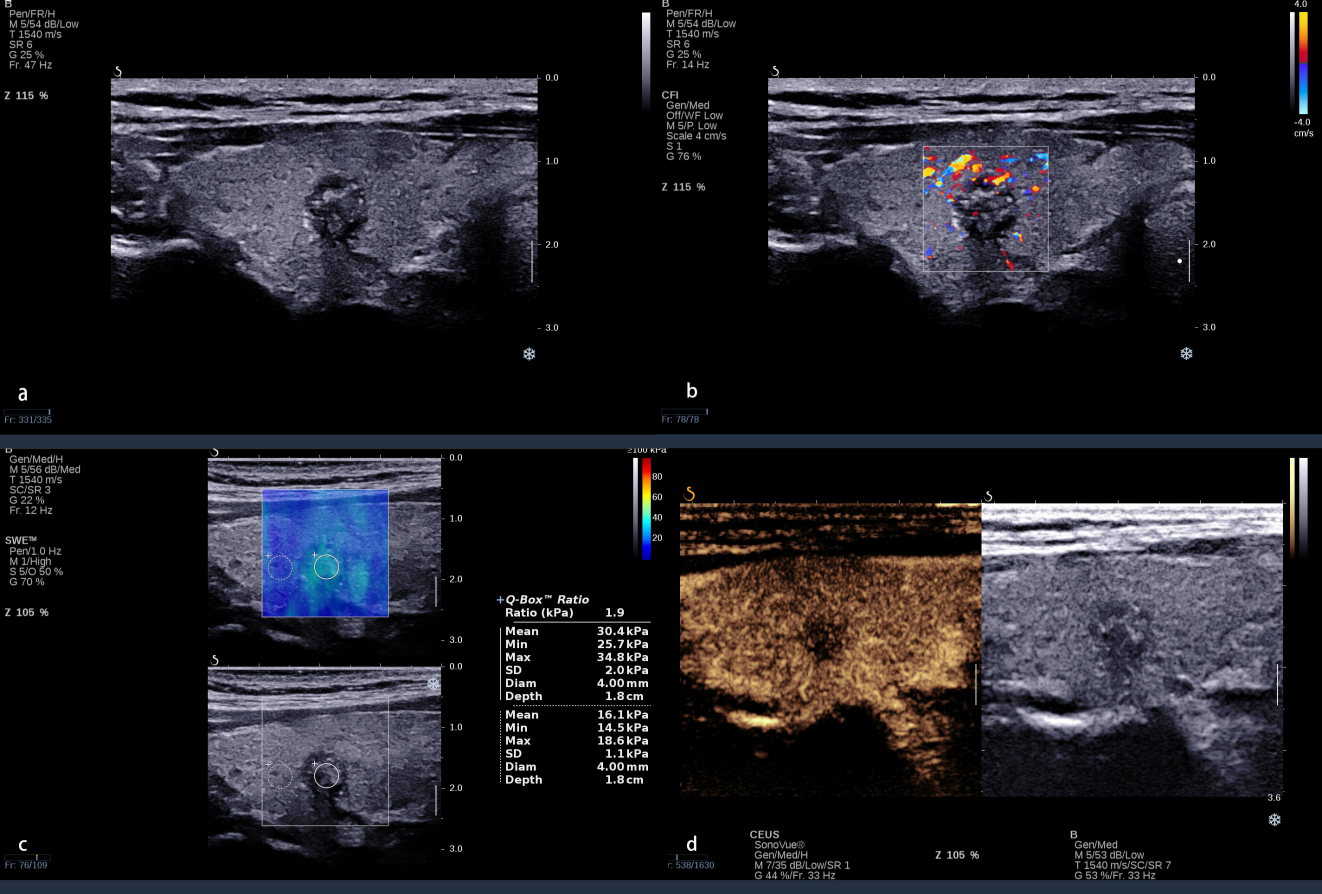
A 38 year-old-woman with a nodule in the right lobe of the thyroid. **(A)** Conventional ultrasound revealed an 8 × 9-mm solid hypo-echoic nodule with an irregular margin, shape that was taller-than-wide, and with punctate echogenic foci; this was categorized ACR TI-RADS 5. **(B)** The nodule presented a spot blood flow signal. **(C)** Quantitative shear wave elastography (SWE) revealed 25.7 kPa for Emin, 30.4 kPa for Emean, 34.8 kPa for Emax, and 1.9 for Eratio. **(D)** Qualitative contrast-enhanced ultrasound (CEUS) of the nodule revealed heterogenous, hypo-enhancement. Even the Emax was <46.8 kPa; the CEUS presented malignant enhancement patterns. The combination of CEUS and SWE recognized this nodule as malignant. The surgical pathology was papillary thyroid carcinoma with Hashimoto’s thyroiditis.

When using Emax alone, there were 10% (five out of 50) benign nodules with Emax >46.8 kPa, which may be because some nodular goiter nodules with fibrosis and calcification could significantly improve the Emax. There were 34.9% (29/83) malignant nodules with Emax <46.8 kPa, and the maximal diameter of 100% (29/29) of these nodules was <10 mm (**Figure 4**). In general, the characteristics of thyroid nodules may be determined already during the initial formation, such as the growth of malignant nodules, the normal follicle damage, the interstitial components being reduced, fibrosis in nodules which increased, and partial nodules presenting calcium and salt deposition. All the above-mentioned features could improve the hardness of thyroid nodules. Therefore, the tissue components gradually change, and the hardness increases, accompanied by the nodules’ increase in size. Thus, the elastography in the differentiation of benign and malignant thyroid nodules might be affected by nodule size. Sengul et al. (38) found that nodule size over 15 mm might strengthen the prediction among high SE scores. Li et al. (39) found that thyroid nodule size affects the optimal Emax cutoff value of SWE. Shang et al. (40) found that Emax was significantly correlated with the size of the nodules. Wang et al. (41) found that conventional ultrasound combined with SWE had higher specificity for nodules smaller than 10 mm and higher sensitivity for nodules larger than 10 mm. Therefore, it was insufficient to identify the characteristics of ACR TI-RADS 4 and 5 category thyroid nodules coexisting with HT, especially for those with maximal diameter <10 mm, which may be related to the small malignant nodules with inconspicuous fibrosis or calcification.

When CEUS was combined with SWE, the sensitivity and accuracy increased compared with using CEUS or SWE alone. Only 6.0% (five out of 83) malignant thyroid nodules were misdiagnosed due to benign enhancement patterns on CEUS and SWE <46.8 kPa. The maximal diameter of all these nodules was <10 mm, including 80% (four out of five) of them which were 5 to 6 mm, which may be caused by inconspicuous neo-vascularization and atypical fibrosis or calcification. Thus, the combination of CEUS and SWE should be carefully used for these small thyroid nodules.

There were several limitations in this study. First, it was a preliminary study in one center with a small sample. Second, qualitative CEUS was used in this study, and no comparison was made with quantitative CEUS. Third, some final pathology results of patients were not available, which may have caused a selection bias of enrolment.

## 5 Conclusion

In conclusion, CEUS and SWE were useful for the differentiation of benign and malignant ACR TI-RADS 4 and 5 category thyroid nodules coexisting with HT. The combination of CEUS and SWE could improve the sensitivity and accuracy compared with using CEUS or SWE alone, which could be a non-invasive, reliable, and useful method to differentiate benign from malignant ACR TI-RADS 4 and 5 category thyroid nodules coexisting with HT, and it might be beneficial to manage patients and improve their prognosis.

## Data availability statement

The raw data supporting the conclusions of this article will be made available by the authors without undue reservation.

## Ethics statement

The studies involving human participants were reviewed and approved by the ethics committee of Yueyang Central Hospital. The patients/participants provided their written informed consent to participate in this study.

## Author contributions

Conception and design: BW, A-JY, X-WC, and CD. Drafting of the article: BW, XO, and JY. Critical revision of the article for important intellectual content: BW, XO, and HZ. All authors contributed to the article and approved the submitted version.

## References

[1] Bomeli SR LeBeau SO Ferris RL. Evaluation of a thyroi d nodule. Otolaryngol Clin North Am (2010) 43(2):229–38. doi: 10.1016/j.otc.2010.01.002 PMC2879398 20510711

[2] Durante C Costante G Lucisano G Bruno R Meringolo D Paciaroni A. The natural history of benign thyroid nodules. JAMA (2015) 313(9):926–35. doi: 10.1001/jama.2015.0956 25734734

[3] Popoveniuc G Jonklaas J. Thyroid nodules. Med Clin North Am (2012) 96(2):329–49. doi: 10.1016/j.mcna.2012.02.002 PMC3575959 22443979

[4] Durante C Grani G Lamartina L Filetti S Mandel SJ Cooper DS. The diagnosis and management of thyroid nodules: A review. JAMA (2018) 319(9):914–24. doi: 10.1001/jama.2018.0898 29509871

[5] Sherman SI Angelos P Ball DW Beenken SW Byrd D. Clark OH et al; national comprehensive cancer network. Thyroid carcinoma J Natl Compr Canc Netw (2005) 3(3):404–57. doi: 10.6004/jnccn.2005.0021 16002006

[6] Noureldine SI Tufano RP. Association of hashimoto's thyroiditis and thyroid cancer. Curr Opin Oncol (2015) 27(1):21–5. doi: 10.1097/CCO.0000000000000150 25390557

[7] Tessler FN Middleton WD Grant EG Hoang JK Berland LL Teefey SA. ACR thyroid imaging, reporting and data system (TI-RADS): White paper of the ACR TI-RADS committee. J Am Coll Radiol (2017) 14(5):587–95. doi: 10.1016/j.jacr.2017.01.046 28372962

[8] Wang D Du LY Sun JW Hou XJ Wang H Wu JQ. Evaluation of thyroid nodules with coexistent hashimoto's thyroiditis according to various ultrasound-based risk stratification systems:A retrospective research. Eur J Radiol (2020) 131:109059. doi: 10.1016/j.ejrad.2020.109059 32739109

[9] Park M Park SH Kim EK Yoon JH Moon HJ Lee HS. Heterogeneous echogenicity of the underlying thyroid parenchyma: How does this affect the analysis of a thyroid nodule? BMC Cancer (2013) 13:550. doi: 10.1186/1471-2407-13-550 24237991 PMC3832886

[10] Singh Ospina N Brito JP Maraka S Espinosa de Ycaza AE Rodriguez-Gutierrez R Gionfriddo MR. Diagnostic accuracy of ultrasound-guided fine needle aspiration biopsy for thyroid malignancy: Systematic review and meta-analysis. Endocrine (2016) 53(3):651–61. doi: 10.1007/s12020-016-0921-x 27071659

[11] Nguyen XV Choudhury KR Eastwood JD Lyman GH Esclamado RM Werner JD. Incidental thyroid nodules on CT: Evaluation of 2 risk-categorization methods for work-up of nodules. AJNR Am J Neuroradiol (2013) 34(9):1812–7. doi: 10.3174/ajnr.A3487 PMC7965620 23557957

[12] Bamber J Cosgrove D Dietrich CF Fromageau J Bojunga J Calliada F. EFSUMB guidelines and recommendations on the clinical use of ultrasound elastography. part 1: Basic principles and technology. Ultraschall Med (2013) 34(2):169–84. doi: 10.1055/s-0033-1335205 23558397

[13] Xu HX Yan K Liu BJ Liu WY Tang LN Zhou Q. Guidelines and recommendations on the clinical use of shear wave elastography for evaluating thyroid nodule1. Clin Hemorheol Microcirc (2019) 72(1):39–60. doi: 10.3233/CH-180452 30320562

[14] Sebag F Vaillant-Lombard J Berbis J Griset V Henry JF Petit P. Shear wave elastography: a new ultrasound imaging mode for the diferential diagnosis of benign and malignant thyroid nodules. J Clin Endocrinol Metab (2010) 95(12):5281–8. doi: 10.1210/jc.2010-0766 20881263

[15] Liu B Liang J Zheng Y Xie X Huang G Zhou L. Two-dimensional shear wave elastography as promising diagnostic tool for predicting malignant thyroid nodules: A prospective single-centre experience. Eur Radiol (2015) 25(3):624–34. doi: 10.1210/jc.2010-0766 25298171

[16] Park AY Son EJ Han K Youk JH Kim JA Park CS. Shear wave elastography of thyroid nodules for the prediction of malignancy in a large scale study. Eur J Radiol (2015) 84(3):407–12. doi: 10.1016/j.ejrad.2014.11.019 25533720

[17] Kim HJ Kwak MK Choi IH Jin SY Park HK Byun DW. Utility of shear wave elastography to detect papillary thyroid carcinoma in thyroid nodules: Efcacy of the standard deviation elasticity. Korean J Intern Med (2018) 34(4):850–7. doi: 10.3904/kjim.2016.326 PMC6610177 29466846

[18] Sidhu PS Cantisani V Dietrich CF Gilja OH Saftoiu A Bartels E. The EFSUMB guidelines and recommendations for the clinical practice of contrast-enhanced ultrasound (CEUS) in non-hepatic applications: Update 2017 (Long version). Ultraschall Med (2018) 39(2):e2–e44. doi: 10.1055/a-0586-1107 29510439

[19] Sengul I Sengul D. Hermeneutics for evaluation of the diagnostic value of ultrasound elastography in TIRADS 4 categories of thyroid nodules. Am J Med Case Rep (2021) 9(11):538–9. doi: 10.12691/ajmcr-9-11-5

[20] Takashima S Matsuzuka F Nagareda T Tomiyama N Kozuka T. Thyroid nodules associated with hashimoto’s thyroiditis: Assessment with US. Radiology (1992) 185:125–30. doi: 10.1148/radiology.185.1.1523294 1523294

[21] Zhang Y Zhou P Tian SM Zhao YF Li JL Li L. Usefulness of combined use of contrast-enhanced ultrasound and TI-RADS classification for the differentiation of benign from malignant lesions of thyroid nodules. Eur Radiol (2017) 27:1527–36. doi: 10.1007/s00330-016-4508-y PMC5334375 27525973

[22] Wang Y Nie F Liu T Yang D Li Q Li J. Revised value of contrast-enhanced ultrasound for solid hypo-echoic thyroid nodules graded with the thyroid imaging reporting and data system. Ultrasound Med Biol (2018) 44:930–40. doi: 10.1016/j.ultrasmedbio.2017.12.018 29472113

[23] Deng J Zhou P Tian SM Zhang L Li JL Qian Y. Comparison of diagnostic efficacy of contrast-enhanced ultrasound, acoustic radiation force impulse imaging, and their combined use in differentiating focal solid thyroid nodules. PloS One (2014) 9(3):e90674. doi: 10.1371/journal.pone.0090674 24594879 PMC3940946

[24] Zhang B Jiang YX Liu JB Yang M Dai Q Zhu QL. Utility of contrast-enhanced ultrasound for evaluation of thyroid nodules. Thyroid (2010) 20(1):51–7. doi: 10.1089/thy.2009.0045 20067379

[25] Cantisani V Consorti F Guerrisi A Guerrisi I Ricci P Di Segni M. Prospective comparative evaluation of quantitative-elastosonography (Q-elastography) and contrast-enhanced ultrasound for the evaluation of thyroid nodules: preliminary experience. Eur J Radiol (2013) 82(11):1892–8. doi: 10.1016/j.ejrad.2013.07.005 23928231

[26] Ma X Zhang B Ling W Liu R Jia H Zhu F. Contrast-enhanced sonography for the identification of benign and malignant thyroid nodules: Systematic review and meta-analysis. J Clin Ultrasound (2016) 44(4):199–209. doi: 10.1002/jcu.22311 26402325

[27] Petrasova H Slaisova R Rohan T Stary K Kyclova J Pavlik T. Contrast-enhanced ultrasonography for differential diagnosis of benign and malignant thyroid lesions: Single-institutional prospective study of qualitative and quantitative CEUS characteristics. Contrast Media Mol Imaging (2022) 2022:8229445. doi: 10.1155/2022/8229445 35542754 PMC9056255

[28] Fu X Guo L Zhang H Ran W Fu P Li Z. “Focal thyroid inferno” on color Doppler ultrasonography: A specific feature of focal hashimoto’s thyroiditis. Eur J Radiol (2012) 81:3319–25. doi: 10.1016/j.ejrad.2012.04.033 22608398

[29] Săftoiu A Gilja OH Sidhu PS Dietrich CF Cantisani V Amy D. The EFSUMB guidelines and recommendations for the clinical practice of elastography in non-hepatic applications: Update 2018. Ultraschall Med (2019) 40(4):425–53. doi: 10.1055/a-0838-9937 31238377

[30] Bojunga J Herrmann E Meyer G Weber S Zeuzem S Friedrich-Rust M. Real-time elastography for the differentiation of benign and malignant thyroid nodules: A meta-analysis. Thyroid (2010) 20(10):1145–50. doi: 10.1089/thy.2010.0079 20860422

[31] Sengul D Sengul I Van Slycke S. Risk stratification of the thyroid nodule with Bethesda indeterminate cytology, category III, IV, V on the one surgeon-performed US-guided fine-needle aspiration with 27-gauge needle, verified by histopathology of thyroidectomy: The additional value of one surgeon-performed elastography. Acta Chir Belg (2019) 119(1):38–46. doi: 10.1080/00015458.2018.1551769 30606092

[32] Zhu L Chen Y Ai H Gong W Zhou B Xu Y. Combining real-time elastography with fine-needle aspiration biopsy to identify malignant thyroid nodules. J Int Med Res (2020) 48(12):300060520976027. doi: 10.1177/0300060520976027 33327813 PMC7747118

[33] Sengul D Sengul I. Reassessing combining real-time elastography with fine-needle aspiration biopsy to identify malignant thyroid nodules. Am J Med Case Rep (2021) 9(11):552–3. doi: 10.12691/ajmcr-9-11-9 PMC7747118 33327813

[34] Moon HJ Sung JM Kim EK Yoon JH Youk JH Kwak JY. Diagnostic performance of gray-scale US and elastography in solid thyroid nodules. Radiol (2012) 262(3):1002–13. doi: 10.1148/radiol.11110839 22357900

[35] Zhang WB Deng WF Mao L He BL Liu H Chen J. Comparison of diagnostic value of SWE, FNA and BRAF gene detection in ACR TI-RADS 4 and 5 thyroid nodules. Clin Hemorheol Microcirc (2022) 81(1):13–21. doi: 10.3233/CH-211280 35068450

[36] Chen Y Dong B Jiang Z Cai Q Huang L Huang H. SuperSonic shear imaging for the differentiation between benign and malignant thyroid nodules: A meta-analysis. J Endocrinol Invest (2022) 45(7):1327–39. doi: 10.1007/s40618-022-01765-y 35229278

[37] Liao LJ Chen HW Hsu WL Chen YS. Comparison of strain elastography, shear wave elastography, and conventional ultrasound in diagnosing thyroid nodules. J Med Ultrasound (2019) 27(1):26–32. doi: 10.4103/JMU.JMU_46_18 31031532 PMC6445028

[38] Sengul D Sengul I Egrioglu E Ozturk T Aydin I Kesicioglu T. Can cut-off points of 10 and 15 mm of thyroid nodule predict malignancy on the basis of three diagnostic tools: i) strain elastography, ii) the Bethesda system for reporting thyroid cytology with 27-gauge fine-needle, and iii) histopathology? J BUON (2020) 25(2):1122–9. 32521915

[39] Li H Kang C Xue J Jing L Miao J. Influence of lesion size on shear wave elastography in the diagnosis of benign and malignant thyroid nodules. Sci Rep (2021) 11(1):21616. doi: 10.1038/s41598-021-01114-8 34732826 PMC8566553

[40] Shang H Wu B Liu Z Liu Y Cheng W. The effectiveness of shear wave elastography in the diagnosis of PTMC. Technol Health Care (2020) 28(2):221–6. doi: 10.3233/THC-191895 31658074

[41] Wang F Chang C Chen M Gao Y Chen YL Zhou SC. Does lesion size affect the value of shear wave elastography for differentiating between benign and malignant thyroid nodules? J Ultrasound Med: Off J Am Institute Ultrasound Med. (2018) 37(3):601–9. doi: 10.1002/jum.14367 28906009

